# Association of Blood‐Based Biomarkers and Malignant Infarction in Severe Anterior Circulation Ischemic Stroke

**DOI:** 10.1002/brb3.71333

**Published:** 2026-03-31

**Authors:** Dominik Lehrieder, Hermann Neugebauer, Alexander M. Kollikowski, Patrick Oeckl, Felipe A. Montellano, Lorenzo Barba, Peter U. Heuschmann, Guido Stoll, Markus Otto, Mirko Pham, Michael K. Schuhmann, Christoph Vollmuth

**Affiliations:** ^1^ Department of Neurology University Hospital Wuerzburg Wuerzburg Germany; ^2^ Department of Neuroradiology University Hospital Wuerzburg Wuerzburg Germany; ^3^ Department of Neurology University Hospital Ulm Ulm Germany; ^4^ German Center for Neurodegenerative Diseases e.V. (DZNE) Site Ulm Ulm Germany; ^5^ Institute For Clinical Epidemiology and Biometry University of Wuerzburg Wuerzburg Germany; ^6^ Department of Neurology Martin‐Luther‐University of Halle‐Wittenberg Halle (Saale) Germany; ^7^ Institute For Medical Data Science University Hospital Wuerzburg Wuerzburg Germany; ^8^ Clinical Trial Centre University Hospital Wuerzburg Wuerzburg Germany; ^9^ Institute of Experimental Biomedicine I University Hospital Wuerzburg Wuerzburg Germany

**Keywords:** blood‐based biomarkers, GFAP, ischemic stroke, malignant infarction, NfL

## Abstract

**Objective:**

To determine the association of serum neurofilament light chain (sNfL) and serum glial fibrillary acidic protein (sGFAP) with the development of a malignant infarction in severe anterior circulation ischemic stroke.

**Methods:**

In this prospective, single‐center cohort study, patients with severe acute ischemic stroke due to large vessel occlusion (LVO) in the anterior circulation were included. Malignant infarction was defined by clinical and neuroradiological parameters. Serum biomarkers levels of NfL and GFAP were determined by ultrasensitive ELISA. Univariable and multivariable logistic regression models, along with receiver operating characteristics (ROC) characteristic analyses, were performed to assess the predictive value of sNfL and sGFAP.

**Results:**

Between 06/2020 and 09/2022, 383 patients were included [54.6% female, median age 79 (interquartile range, (IQR) 69–84) years, median NIHSS Score on admission 14 (IQR 9–17)]. Biomarker serum levels were measured at a median of 36 [IQR 23–61] hours after symptom onset. Malignant infarction occurred in 83 patients (21.7%). Both sNfL and sGFAP levels were significantly elevated in patients with compared to those without malignant infarction (sNfL: 208 pg/ml vs. 96 pg/ml, *p* < 0.001; sGFAP: 36.6 ng/ml vs. 4.1 ng/ml, *p* < 0.001). After adjusting for clinical and radiological parameters, sNfL and sGFAP remained independent predictors for malignant infarction (sNfL: OR 2.91 [1.55–5.45], *p* < 0.001; sGFAP: 3.49 [1.80–6.75], *p* < 0.001). The inclusion of both biomarkers in prediction models significantly improved their predictive value.

**Conclusions:**

Serum NfL and GFAP independently predict malignant infarction in severe anterior circulation ischemic stroke, albeit with only a low effect size in models with established prognostic factors. Further studies investigating earlier and serial biomarker measurement are required to improve prognostic models supporting risk stratification and decision making.

**Study registration:**

DRKS00022064.In severe anterior circulation ischemic stroke due to LVO, sNfL and sGFAP levels were significantly elevated in patients who developed malignant infarction. Both biomarkers independently predicted malignant infarction after adjustment for clinical and radiological factors. Nevertheless, the additional predictive value beyond established prognostic parameters was modest.

## Graphical abstract

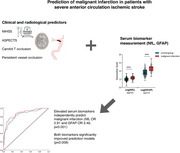

## 1 Introduction

LVO accounts for up to 46% of acute ischemic strokes (AIS), with the majority occurring in the anterior circulation (Smith et al. 2009). While widespread implementation of mechanical thrombectomy marks a significant advance in reducing disability among these patients (Goyal et al. 2016), approximately one in five patients develops malignant cerebral edema following the procedure (Zhang et al. 2023). It is still uncertain which patients are likely to experience swelling and which are not. Given the potential for transtentorial herniation, malignant infarction represents a life‐threatening condition, underscoring the critical importance of early detection among individuals at elevated risk (Hacke et al. 1996). In clinical practice, imaging and clinical parameters, including age, NIHSS Score on admission, hypoattenuation >50% of middle cerebral artery (MCA) territory on computed tomography and vessel revascularization, have been studied for this purpose, whereas blood‐based biomarkers, which can easily be obtained, remain still less explored (Wu et al. 2018; Ng et al. 2017). sNfL and sGFAP are complementary biomarkers that reflect neuronal injury and astrocyte damage, respectively. Both have been demonstrated to predict functional outcome after severe ischemic stroke in recent studies (Vollmuth et al. 2024; Pujol‐Calderón et al. 2022; Correia et al. 2022). The aim of this study was to investigate the association of sNfL and sGFAP with the development of malignant infarction in severe anterior circulation ischemic stroke. Additionally, we sought to assess the incremental value of these blood‐based biomarkers when added to established prognostic models (Jo et al. 2017).

## 2 Methods

### 2.1 Standard Protocol Approvals, Registrations, and Patient Consents

All patients analyzed participated in a single‐center, prospective study conducted at a tertiary neurovascular center in Germany. The study was registered in the German Registry for Clinical Studies (DRKS00022064) and approved by the local ethics committee of the University of Wuerzburg (reference n. 05/20‐am). Written informed consent was obtained from all patients or legal representatives. Supplementary Material.

### 2.2 Patients

From June 2020 to September 2022, patients presenting with severe AIS, as defined by a National Institutes of Health Stroke Scale (NIHSS) Score of 6 points or more and/or with an indication for mechanical thrombectomy, were enrolled in the prospective, single‐center cohort study “Würzburg stroke cohort”. The present analysis included patients who met the following criteria: (1) a MCA infarction was present; (2) a large cerebral vessel was occluded; and (3) serum biomarkers were determined during the patient's hospitalization. The exclusion criteria included age below 18 years, inadequate German language proficiency, and participation in another clinical trial.

### 2.3 Clinical Parameters

After study enrollment demographic and clinical variables were collected and entered into a centralized database. Baseline characteristics include age, gender, pre‐stroke modified Rankin Scale (mRS) Score and vascular comorbidities (arterial hypertonus, atrial fibrillation, heart failure, diabetes mellitus). Stroke severity was assessed on admission, after 24, 48, 72 h, and at discharge using the NIHSS Score. The functional outcome was evaluated using the mRS on discharge and after three months by telephone interview by a blinded rater. Additionally, data was collected on acute stroke treatment, including intravenous thrombolysis and endovascular thrombectomy, as well as the recanalization status based on the expanded treatment in cerebral ischemia (eTICI) score and performance of decompressive hemicraniectomy during the course of the disease was recorded. Radiological variables, including the Alberta stroke program early CT score (ASPECTS) on admission, maximal midline shift in further course, additional anterior cerebral artery infarction and vessel occlusion localization were obtained by an experienced neuroradiologist. Furthermore, the time from symptom onset to recanalization therapy and from symptom onset to blood drawn was recorded. In instances where the time of stroke onset was unknown, or where the patient experienced a wake‐up stroke, the time from when the patient was last known to be well was utilized.

### 2.4 Definition of Malignant Infarction

Malignant infarction of the MCA was defined by fulfilling the neuroradiological definition (affecting at least two‐thirds of the MCA territory or more than half of the MCA territory and inclusion of the basal ganglia) in combination with the presence of a severe clinical syndrome (NIHSS score ≥ 15 points within 72 h after symptom onset) (Huttner and Schwab 2009).

### 2.5 Serum Biomarker Measurement

Blood samples were collected from the antecubital or femoral vein on the morning following enrollment. They were centrifuged at 2500 × g for 10 min to separate serum, which was then snap‐frozen and stored at −80°C until analysis. Biomarker measurements were conducted following the manufacturers´ protocols and without access to the patients´ clinical data to ensure blinding. Specifically, serum GFAP concentrations were quantified using a commercially available digital immunoassay on the HD‐X Simoa platform (Quanterix Inc., Lexington, USA). Serum NfL levels were assessed using a commercial kit designed for the automated ELLA microfluidic system (BioTechne, Minneapolis, USA).

### 2.6 Statistical Analysis

The statistical analyses were conducted using the SPSS Inc., Chicago, IL (SPSS 29), GraphPad Software, La Jolla, USA (GraphPad 10), and R Foundation, Vienna, Austria (R 4.4.2) software packages. The level of significance was defined as *p* < 0.05. Univariable analyses were performed to compare malignant infarction group and the control group. The Student's *t* test, Mann‐Whitney *U* test, or χ^2^ test were employed as appropriate for all variables. A mRs score ≤ 4 after three months was defined as an acceptable functional outcome (Lehrieder et al. 2021; Neugebauer et al. 2017). Data was transferred using log10 to reduce the skewness of the biomarker values. However, to present the results in a clear and accessible manner, medians and IQRs are presented on the original scale. Furthermore, Spearman's correlation analysis was performed to investigate the influence of age and renal function on sNFL and sGFAP, as previously described (Abu‐Rumeileh et al. 2022). Using ROC analysis, we calculated serum biomarker (sNfL and sGFAP) threshold by areas under the curve (AUCs) with 95% confidence intervals (95% CIs). The optimal cutoff values discriminating between both groups were chosen to maximize the Youden Index (sensitivity + specificity – 1) (Youden 1950). In light of the pivotal role of high sensitivity to identify patients at risk of malignant swelling, a cutoff value was determined with both a high degree of sensitivity—exceeding 95%—and acceptable specificity. Furthermore, with the objective of selecting patients who were at low risk for malignant infarction, a cutoff value with a specificity > 95% and acceptable sensitivity was calculated. To identify the independent prognostic value of both serum biomarkers on malignant infarction, they were entered as continuous (log10 value) and categorial (above cutoff value using Youden Index) variables separately into multivariable binary logistic regression model using backward selection. Adjustments were made for clinically relevant variables that differed significantly between groups, or that have been identified as influential on the measurement of both biomarkers (age, kidney function, symptom onset to blood drawn) or had previously been established as prognostic (NIHSS score and ASPECTS on admission, recanalization status) (Jo et al. 2017). As this is a post‐hoc analysis of a prospective, observational study, no sample size calculation was performed. The number of covariates included in the multivariable regression model was prespecified and limited according to the study cohort and the number of outcome events, in order to ensure an adequate events‐per‐variable ratio and maintain model stability while minimizing the risk of overfitting. Prior to analysis, the absence of critical multicollinearity between all parameters was verified through the implementation of Spearman's correlation analysis. Results were expressed as odds ratios (ORs) with 95% CIs. Significant parameters of the final stage of backward selection were entered into prognostic models. Area under the ROC Characteristic (AUROC) curve was analyzed for each model to investigate the discriminative value for malignant infarction. The incremental value of both biomarkers was evaluated by analyzing the improvement of discrimination using the DeLong test (DeLong et al. 1988).

## 3 Results

**FIGURE 1:**
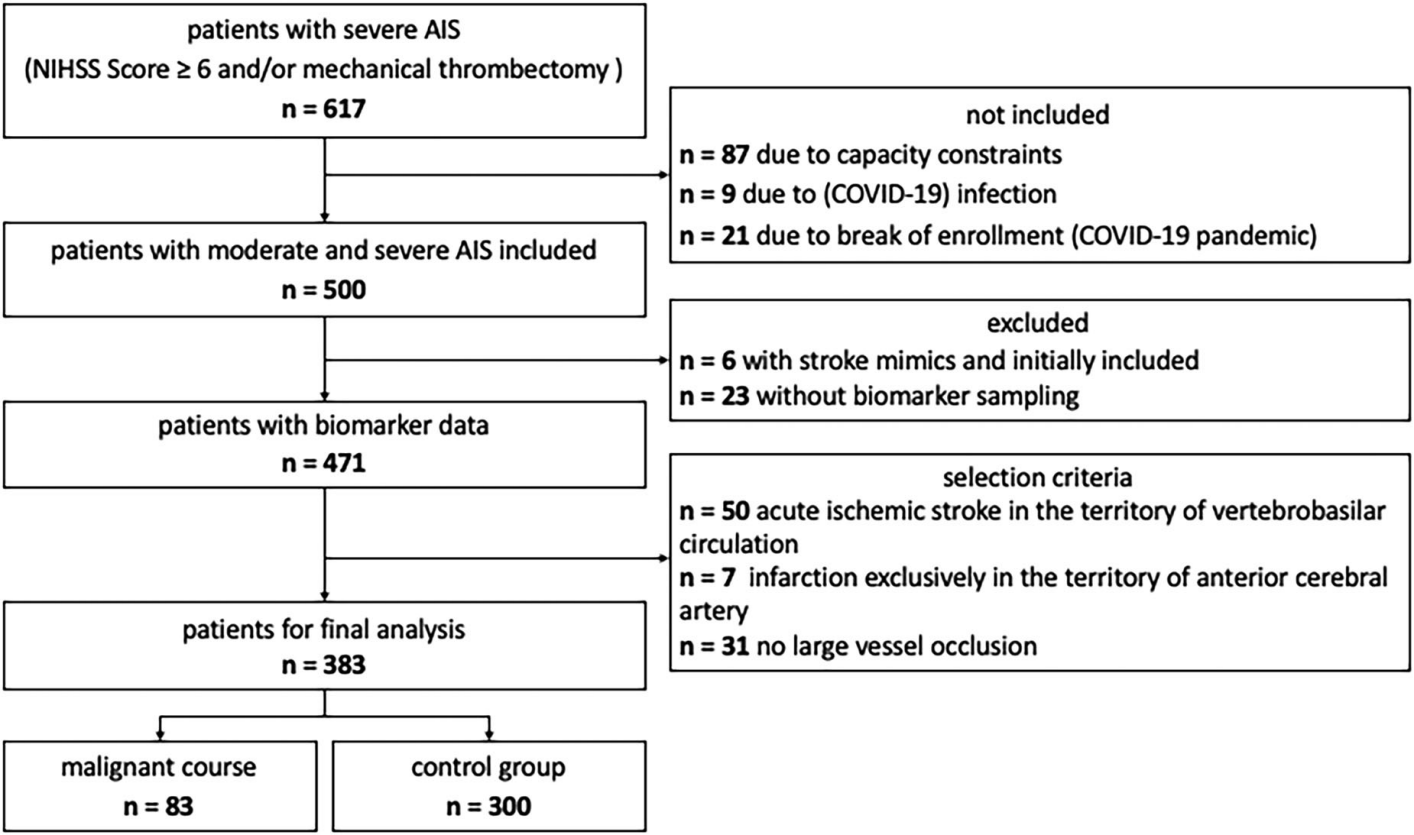
Flowchart of study population.

Between June 2020 and September 2022, a total of 617 patients with severe acute ischemic stroke were admitted to our tertiary care center (University Hospital Wuerzburg, Wuerzburg, Germany). However, due to a temporary pause in enrollment during the COVID‐19 pandemic (December 2020–January 2021), 21 stroke patients treated in that period were not included in the study. Additionally, 87 patients were excluded due to capacity constraints, and nine patients were excluded due to COVID‐19 infection. Of the remaining 500 patients initially enrolled, six were later excluded as they were determined to have stroke mimics. Blood samples were not available for 23 patients. Further, 50 patients presented with an infarction in the vertebrobasilar territory, 31 showed no evidence of LVO and seven had an infarction exclusively affecting the anterior cerebral artery and were excluded. Consequently, a total of 383 patients were included in the analysis (Figure 1).

Of the 383 patients included in this analysis, 209 (54%) were female, with a median IQR age of 79 (69‐84) years. The median (IQR) on the NIHSS Score was 13.5 (9–17), and 54 (14%) had an ASPECT Score on admission ≤ 5 points. Most patients (83%) underwent mechanical thrombectomy, while 153 (40%) received systemic thrombolysis and 117 (31%) patients received both recanalizing therapies. The median (IQR) duration from symptom onset to recanalization therapy was 4.3 (2.3–7.9) h, and 36.3 (23.1–60.9) h from symptom onset to blood draw.

**FIGURE 2:**
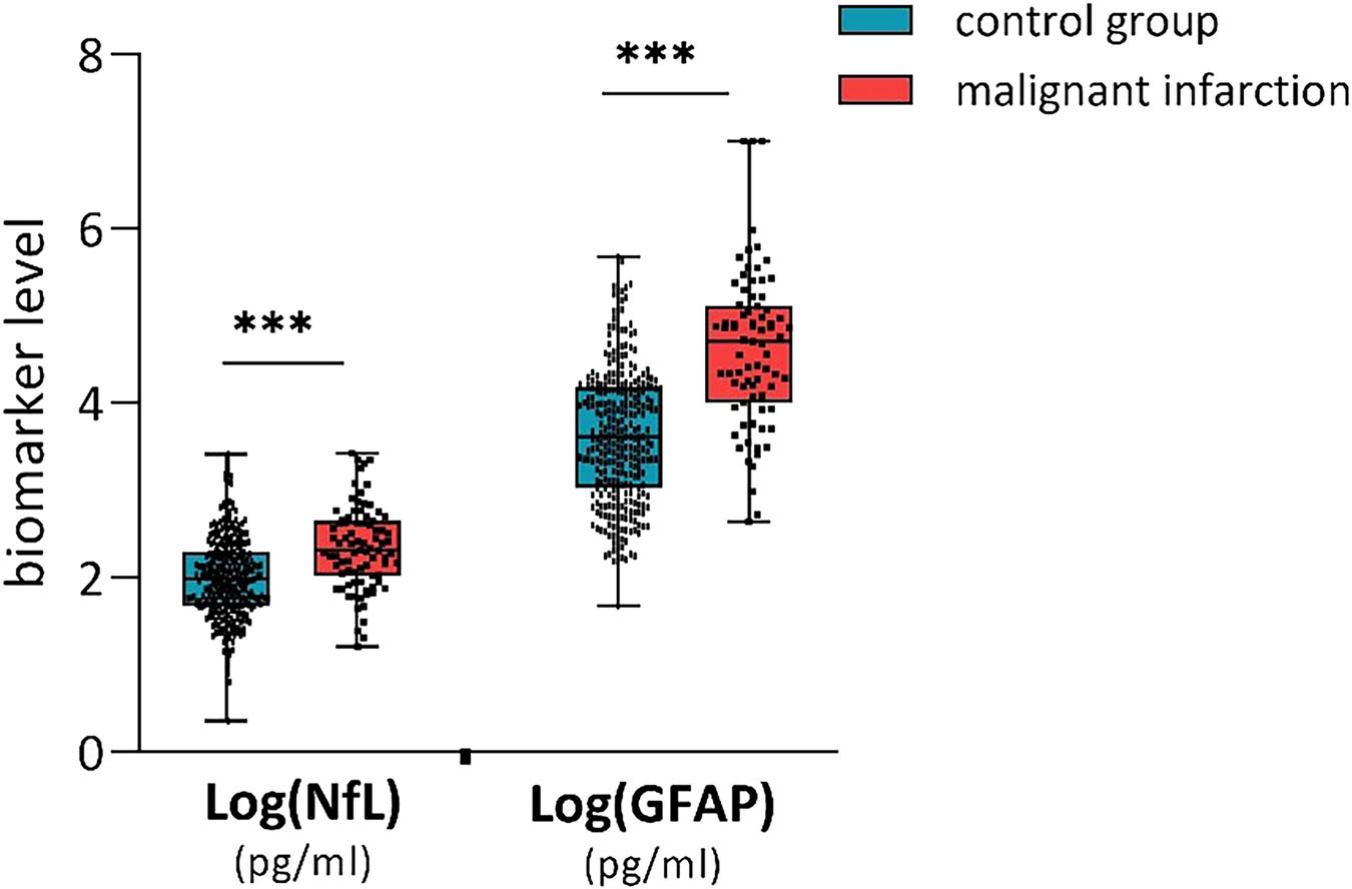
Biomarker level according to malignant infarction compared to control group. *** *p* value <0.001.

A malignant infarction was identified in 83 patients (21.7%) among the total study cohort. The demographic and clinical characteristics of both groups are summarized in Table 1. Patients who developed a malignant infarction exhibited a higher stroke severity on admission (NIHSS Score: 16 [14–19] vs. 12 [8–16], *p* < 0.001) and greater prevalence of a lower ASPECT score in acute imaging (ASPECTS ≤ 5: 42.1% vs. 6.3%, *p* < 0.001). Furthermore, proximal occlusion was more frequently observed in the anterior circulation, with a higher prevalence of carotid‐T (25.3% vs. 7.3%, *p* < 0.001) and M1 segment (72.3% vs. 59.7, *p* = 0.036) occlusions. While endovascular treatment was indicated with equal frequency, vessel occlusion persisted more frequently in patients with a malignant infarction (41.0% vs. 13.3%, *p* < 0.001). 10 patients with a malignant infarction underwent hemicraniectomy (12.2%). At the three‐month follow‐up, 57 (70.4%) patients with a malignant infarction had died, while only 19 (23.4%) had an acceptable outcome (mRS ≤ 4), compared to 74 (25.3%) and 202 (67.3%) of the control group (*p* < 0.001). The blood draw was conducted at a slightly later point in time following the onset of symptoms in patients with malignant infarction (40.3 [26.7–72.1] h vs. 36 [22.6–59] h, *p* = 0.041). Patients with a malignant infarction exhibited elevated serum biomarker levels of neurofilament light chain (208 [104–445] pg/ml vs. 96 [48–199] pg/ml, *p* < 0.001) and sGFAP (36.6 [8.3–117.9] ng/ml vs. 4.1 [1.1–15.1] ng/ml, *p* < 0.001), as illustrated in Figure 2. No significant differences between study groups were observed with regard to age, gender, creatinine, the presence of cardiovascular comorbidities, pre‐stroke morbidity, imaging modality, or stroke etiology.

**TABLE 1:** Sociodemographic and clinical characteristics of study cohort.

—	Control (*N* = 300)	Malignant infarction (*N* = 83)	Overall (*N* = 383)	*p* value
Age, M (IQR)	79 (69–84)	79 (63–85)	79 (69–84)	0.68
Female, *N* (%)	166 (55.3)	43 (51.8)	209 (54.6)	0.57
Prestroke mRS, M (IQR)	0 (0–2)	0 (0–2)	0 (0–2)	0.32
Atrial fibrillation, *N* (%)	150 (50.0)	39 (47.0)	189 (49.3)	0.63
Arterial hypertension, *N* (%)	200 (66.7)	54 (65.1)	254 (66.3)	0.78
Diabetes mellitus, *N* (%)	57 (19.0)	16 (19.3)	73 (19.1)	0.96
Chronic heart failure, *N* (%)	39 (13.0)	17 (20.5)	56 (14.6)	0.09
Systemic thrombolysis, *N* (%)	125 (41.7)	28 (33.7)	153 (39.9)	0.19
Mechanical thrombectomy, *N* (%)	256 (85.3)	62 (74.7)	318 (83.0)	0.022
Any recanalization therapy, *N* (%)	289 (96.3)	65 (78.3)	354 (92.4)	<0.001
Symptom‐onset to mechanical thrombectomy in h, M (IQR)	4.2 (2.7–8.9)	4.9 (2.9–12.0)	4.3 (2.7–9.7)	0.29
Symptom‐onset to systemic thrombolysis in h, M (IQR)	1.6 (1.3–2.5)	1.7 (1.1–2.5)	1.6 (1.3–2.5)	0.92
Symptom‐onset to first recanalization therapy in h, M (IQR)	3.8 (2.2–7.1)	4.6 (2.7–11.0)	4.0 (2.3–7.9)	0.18
eTICI score (treated with mechanical thrombectomy, *N* = 318), *N* (%)				<0.001
0	10 (3.9)	9 (14.5)	19 (6.0)	
1	3 (1.2)	1 (1.6)	4 (1.3)	
2a	12 (4.7)	6 (9.7)	18 (5.7)	
2b	112 (43.8)	37 (59.7)	149 (46.9)	
2c	56 (21.9)	2 (3.2)	58 (18.2)	
3	63 (24.3)	7 (11.3)	70 (22.0)	
Persistent large vessel occlusion after acute treatment, *N* (%)	40 (13.3)	34 (41.0)	74 (19.3)	<0.001
Hemicraniectomy, *N* (%)	0 (0.0)	10 (12.2)	10 (2.6)	<0.001
NIHSS admission, M (IQR)	12 (8–16)	16 (14–19)	13.5 (9–17)	<0.001
NIHSS 24 h, M (IQR)	8.5 (4–16)	27 (19–36)	12 (5–21)	<0.001
NIHSS 48 h, M (IQR)	7 (2–14)	27 (18.5–36)	11 (4–18)	<0.001
NIHSS 72 h, M (IQR)	6 (2–13)	29 (18.25–36)	9 (2–16)	<0.001
NIHSS discharge, M (IQR)	3 (1–9)	14.5 (12–17.25)	4 (1–11)	<0.001
Midline shift at follow up imaging, *N* (%)	35 (11.7)	62 (76.5)	97 (25.5)	<0.001
Maximum midline shift at follow up imaging, in mm, M (IQR)	0 (0–0)	4 (2–7)	0 (0–2)	<0.001
ASPECTS on admission, *N* (%)				
0–2	1 (0.3)	7 (8.4)	8 (2.1)	<0.001
3–5	18 (6.0)	28 (33.7)	46 (12.0)	
6–10	281 (93.7)	48 (57.8)	329 (85.9)	
Vessel ccclusion on admission				
M1, *N* (%)	179 (59.7)	60 (72.3)	239 (62.4)	0.036
M2, *N* (%)	92 (30.7)	7 (8.4)	99 (25.8)	<0.001
ACC, *N* (%)	2 (0.7)	2 (2.4)	4 (1.0)	0.17
ACI, *N* (%)	54 (18.0)	22 (26.5)	76 (19.8)	0.09
Carotid‐T, *N* (%)	22 (7.3)	21 (25.3)	43 (11.2)	<0.001
Additional ACA infarction on admission, *N* (%)	12 (4.0)	9 (10.8)	21 (5.5)	0.015
Etiology (TOAST criteria), *N* (%)				
Large‐artery atherosclerosis	48 (16.0)	11 (13.3)	59 (15.4)	0.73
Cardioembolism	150 (50.0)	38 (45.8)	188 (49.1)	
Other determined etiology	7 (2.3)	2 (2.4)	9 (2.3)	
Undetermined etiology	85 (28.3)	30 (36.1)	115 (30.0)	
Concurrent etiology	10 (3.3)	2 (2.4)	12 (3.1)	
mRS 3‐months, *N* (%)				
0	31 (10.6)	0 (0.0)	31 (8.3)	<0.001
1	49 (16.7)	0 (0.0)	49 (13.1)	
2	16 (5.5)	2 (2.5)	18 (4.8)	
3	57 (19.5)	4 (4.9)	61 (16.3)	
4	49 (16.7)	13 (16.0)	62 (16.6)	
5	17 (5.8)	5 (6.2)	22 (5.9)	
6	74 (25.3)	57 (70.4)	131 (35.0)	
Symptom‐onset to blood draw in h, M (IQR)	36 (22.6–59)	40.3 (26.7–73.1)	36.3 (23.1–60.9)	0.041
Serum neurofilament light chain in pg/ml, M (IQR)	96 (48–199)	208 (104–445)	113 (53–154)	<0.001
Serum glial fibrillary acidic protein in ng/ml, M (IQR)	4.1 (1.1–15.1)	36.6 (8.3–117.9)	7.3 (1.6–22.4)	<0.001
Creatinine in mg/dl, M (IQR)	0.92 (0.77–1.21)	0.96 (0.81–1.20)	0.93 (0.78–1.21)	0.44

Spearman´s correlation showed a very weak positive relationship of sNfL with age (*p* < 0.001, *r* = 0.18 [95% CI 0.08 to 0.28]) and a weak positive relationship with serum creatinine (*p* < 0.001, *r* = 0.23 [0.13 to 0.32]), whereas no relationship was found for sGFAP for both parameters. In addition, we found a significant moderate positive correlation between serum NFL and GFAP (*p* < 0.001, *r* = 0.49 [0.40 to 0.56]). Regarding the prognostic parameters, serum GFAP showed a weak positive relationship with persistent occlusion (*p* < 0.001, *r* = 0.20 [0.10 to 0.30], a moderate positive relationship with NIHSS score on admission (*p* < 0.001, *r* = 0.36 [0.26 to 0.44]) and a strong negative relationship with ASPECTS on admission (*p* < 0.001, *r* = –0.56 [–0.63 to –0.49]). Serum NFL showed a very weak positive relationship with persistent occlusion (*p* = 0.004, *r* = 0.15 [0.04 to 0.25]), a weak positive relationship with NIHSS score on admission (*p* < 0.001, *r* = 0.21 [0.11 to 0.31]) and a weak negative relationship with ASPECTS on admission (*p* < 0.001, *r* = –0.24 [–0.33 to –0.13]).

The ROC analysis of both serum biomarkers was performed to predict malignant infarction. For NFL, a cutoff value of 138.5 pg/ml was determined using the Youden index, resulting in a sensitivity of 68.7% and a specificity of 65.0% (AUC 0.70 [0.64–0.77], *p* < 0.001). Further cutoff values were identified, including 43.5 pg/ml, yielding a higher sensitivity of 95.2% with a specificity of 22.0%, and 643 pg/ml, providing a higher specificity of 95.5% with a sensitivity of 14.5%. Similarly, for sGFAP, a cutoff value of 17.1 ng/ml was calculated using the Youden index, yielding a sensitivity of 68.4% and a specificity of 79.3% (AUC 0.80 [0.75–0.86], *p* < 0.001). Lowering the cutoff to 1.9 ng/ml enhanced sensitivity to 96.2% at the expense of specificity (33.0%), while increasing the cutoff to 108 ng/ml improved specificity to 96.5% with a sensitivity of 14.5%.

The final stages of backward stepwise multivariable logistic regression analysis are illustrated in Table 2. Following adjustments for established clinical and radiological parameters (NIHSS Score on admission, ASPECT Score on admission, carotid T‐occlusion, and persistent vessel occlusion) as well as parameters that are known to influence biomarker measurement (age, creatinine, time from symptom onset to blood drawn), both sNFL and sGFAP were found to be independent predictors of a malignant infarction (sNFL log: OR 3.17 [1.62–6.18], *p* < 0.001, sNFL cutoff: OR 2.91 (1.55–5.45), *p* < 0.001, and sGFAP log: OR 2.65 [1.66–4.21], *p* < 0.001, sGFAP cutoff: 3.49 (1.80–6.75), *p* < 0.001).

**TABLE 2:** Final stages of backward stepwise multivariable logistic regression analysis to predict malignant infarction.

—	Crude OR^a^ (95% CI)	Adjusted OR^b^ (95% CI)	*p* value
Neurofilament light chain (log)	4.41 (2.57–7.58)	3.17 (1.62–6.18)	<0.001
Persistent large vessel occlusion after acute treatment	4.51 (2.60–7.82)	2.85 (1.41–5.78)	0.004
ASPECT Score on admission	0.49 (0.41–0.59)	0.57 (0.47–0.70)	<0.001
NIHSS Score on admission	1.13 (1.08–1.19)	1.08 (1.03–1.14)	0.005
Carotid‐T occlusion on admission	4.28 (2.22–8.27)	2.86 (1.26–6.47)	0.012
Neurofilament light chain (cutoff > 138.5 pg/ml)	4.07 (2.42–6.86)	2.91 (1.55–5.45)	<0.001
Persistent large vessel occlusion after acute treatment	4.51 (2.60–7.82)	3.03 (1.49–6.17)	0.002
ASPECT Score on admission	0.49 (0.41–0.59)	0.57 (0.47–0.70)	<0.001
NIHSS Score on admission	1.13 (1.08–1.19)	1.08 (1.02–1.14)	0.005
Carotid‐T occlusion on admission	4.28 (2.22–8.27)	3.16 (1.39–7.17)	<0.001
Glial fibrillary acidic protein (log)	4.49 (3.03–6.64)	2.65 (1.66–4.21)	<0.001
Persistent large vessel occlusion after acute treatment	4.51 (2.60–7.82)	3.46 (1.70–7.05)	<0.001
ASPECT Score on admission	0.49 (0.41–0.59)	0.68 (0.55–0.84)	<0.001
NIHSS Score on admission	1.13 (1.08–1.19)	1.07 (1.01–1.13)	0.020
Carotid‐T occlusion on admission	4.28 (2.22–8.27)	3.07 (1.28–7.39)	0.012
Glial fibrillary acidic protein (cutoff > 17.1 ng/ml)	8.29 (4.78–14.38)	3.53 (1.82–6.82)	<0.001
Persistent large vessel occlusion after acute treatment	4.51 (2.60–7.82)	3.45 (1.68–7.06)	<0.001
ASPECT Score on admission	0.49 (0.41–0.59)	0.64 (0.52–0.79)	<0.001
NIHSS Score on admission	1.13 (1.08–1.19)	1.08 (1.02–1.15)	0.006
Carotid‐T occlusion on admission	4.28 (2.22–8.27)	3.22 (1.38–7.49)	0.007

**^Notes: a^ :**All *p* values < 0.001.

^b^ After adjustment for age, creatinine, time between symptom onset to blood draw, Carotid‐T occlusion on admission, NIHSS on admission, ASPECTS on admission, and persistent vessel occlusion after acute treatment.

**FIGURE 3:**
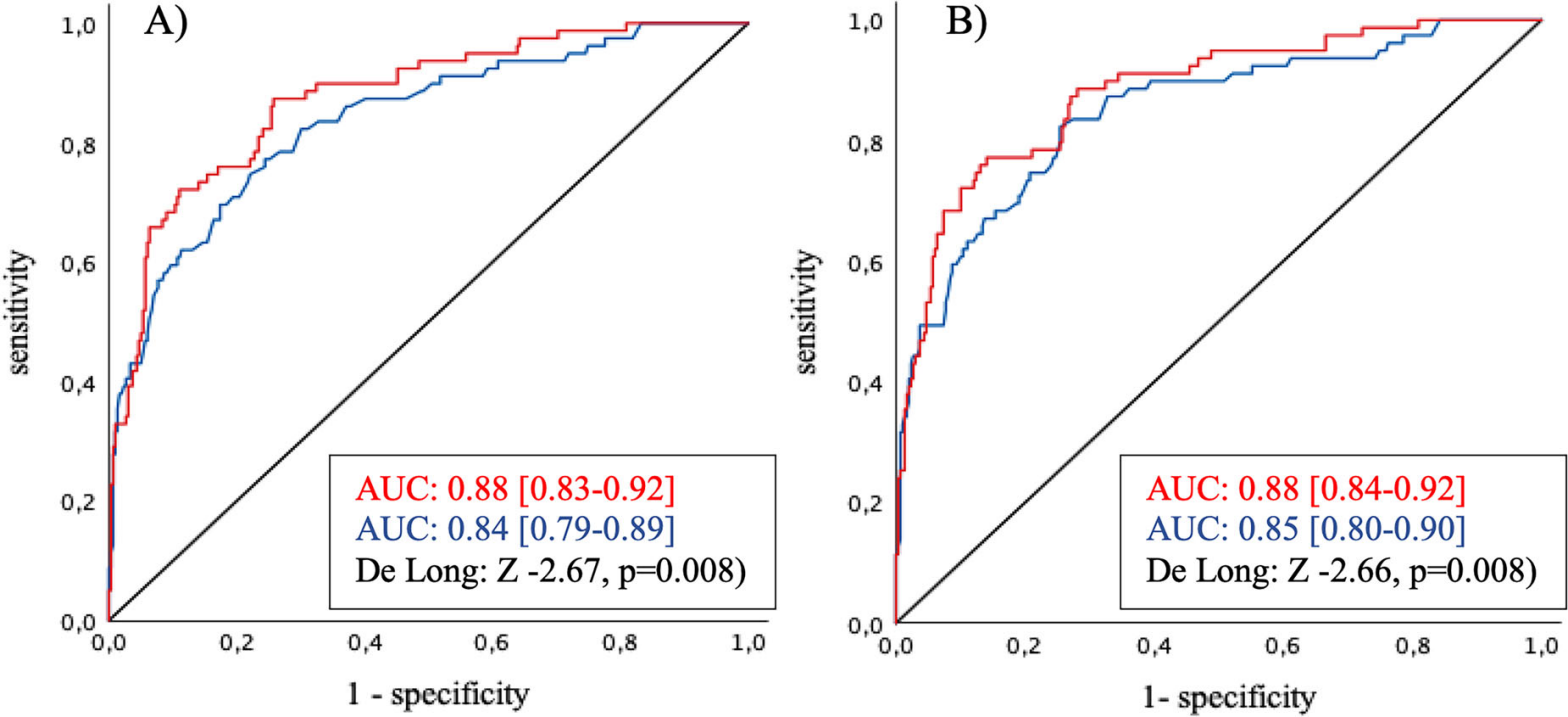
ROC curve of predicted probabilities for (A) ASPECT Score on admission / NIHSS Score on admission / persistent occlusion after acute treatment (blue) vs. ASPECT Score on admission /NIHSS Score on admission / persistent occlusion after acute treatment/ sNFL / sGFAP (red); and (B) ASPECT Score on admission / NIHSS Score on admission / persistent occlusion after acute treatment / carotid T‐occlusion on admission (blue) vs. ASPECT Score on admission / NIHSS Score on admission / persistent occlusion after acute treatment / carotid T‐occlusion on admission / sNFL / sGFAP (red).

Incorporating measurement of both biomarkers in a prediction model with two of the additional independent predictors (NIHSS Score on admission, ASPECT Score on admission, carotid T‐occlusion and persistent occlusion after acute treatment) as illustrated in Table 3 significantly improved model performance. DeLong test results demonstrated this benefit across all tested combinations: ASPECTS/NIHSS vs. ASPECTS/NIHSS + both biomarkers (Z: –2.91, *p* = 0.004); ASPECTS/persistent occlusion vs. ASPECTS/persistent occlusion + both biomarkers (Z: –2.99, *p* = 0.003); NIHSS/carotid T‐occlusion vs. NIHSS/carotid T‐occlusion + both biomarkers (Z: –3.38, *p* < 0.001); NIHSS/persistent occlusion vs. NIHSS/persistent occlusion + both biomarkers (Z: –4.19, *p* < 0.001); and carotid T‐occlusion/persistent occlusion vs. carotid T‐occlusion/persistent occlusion + both biomarkers (Z: –5.32, *p* < 0.001). Additionally, integrating both serum biomarkers enhanced a prediction model containing ASPECT Score, NIHSS Score, and persistent occlusion (AUC: 0.88 [0.83–0.92] vs. 0.84 [0.79–0.89], Z: –2.67, *p* = 0.008) and even a comprehensive model including all four predictors (AUC: 0.88 [0.84–0.92] vs. 0.85 [0.80–0.90], Z: –2.66, *p* < 0.008) (Figure 3). Furthermore, the incorporation of a single biomarker was sufficient in all calculated models, irrespective of the specific biomarker selected with only one exception. Regarding NIHSS/carotid T‐occlusion, the solitary measurement of sNFL was not beneficial (Z: ‐1.47, *p* = 0.14). By contrast, regarding NIHSS/persistent occlusion, adding sGFAP was superior to sNFL (Z: –2.43, *p* = 0.02).

**TABLE 3:** AUROC with confidence intervals with and without blood‐based biomarkers and level of significance of the DeLong test for the comparison with the model without biomarkers.

Model	w/o biomarker	+sNFL	+sGFAP	+sNFL/sGFAP
ASPECTS on admission × NIHSS on admission	0.81 (0.76–0.87)	0.85 (0.80–0.89)^*^	0.86 (0.81–0.89) **	0.86 (0.81–0.91) **
ASPECTS on admission× persistent occlusion after acute treatment	0.82 (0.76–0.88)	0.86 (0.81–0.90)^**^	0.87 (0.82–0.91) **	0.87 (0.83–0.92) **
NIHSS on admission × carotid T‐occlusion on admission	0.75 (0.69–0.81)	0.78 (0.72–0.83)^ns^	0.84 (0.79–0.89)***	0.84 (0.78–0.89) ***
NIHSS on admission × persistent occlusion after acute treatment	0.76 (0.69–0.82)	0.80 (0.75–0.86)^**^	0.85 (0.80–0.90)***	0.85 (0.80–0.90) ***
persistent occlusion after acute treatment × carotid T‐occlusion on admission	0.70 (0.63–0.77)	0.79 (0.74–0.85)^***^	0.85 (0.80–0.90)***	0.85 (0.80–0.90) ***
ASPECTS on admission × NIHSS on admission x persistent occlusion after acute treatment	0.84 (0.79–0.89)	0.86 (0.82–0.91)*	0.87 (0.83–0.92)*	0.88 (0.83–0.92) **
ASPECTS on admission × NIHSS on admission × persistent occlusion after acute treatment x carotid T‐occlusion on admission	0.85 (0.80–0.90)	0.867 (0.82–0.92)*	0.88 (0.83–0.92)*	0.88 (0.84–0.92) **

*Note*: DeLong test: * *p* < 0.05; ** *p* < 0.01; and *** *p* < 0.001; ^ns^ not significant.

## 4 Discussion

Due to the life‐threatening nature of space‐occupying middle cerebral artery infarction, it is imperative to identify patients at high risk for progression to malignant infarction. The present study investigates blood‐based neurofilament light chain and sGFAP as predictors of malignant infarction in severe anterior circulation infarction. Both serum biomarkers were found to be independent predictors of malignant outcome and their prognostic value exceeded that of established clinical and radiological parameters.

Given the severity of the neurological syndrome and the low proportion of advanced early infarct signs on admission, this is a representative group of stroke patients qualifying for endovascular treatment. At around 20%, the frequency of malignant infarction is within the expected range of thrombectomy patients (Zhang et al. 2023). However, due to the advanced age of the population, with a median of approximately 80 years, only 10 patients (12%) underwent subsequent hemicraniectomy. Consequently, mortality and functional long‐term outcome aligned with the findings of conservative control groups in the European randomised controlled hemicraniectomy trials (Vahedi et al. 2007; Jüttler et al. 2014).

An increase in sNfL > 139 pg/ml or sGFAP > 17 ng/ml increases the risk of malignant infarction by a factor of 2.9 and 3.5, respectively. Although, these cut‐off values were calculated with regard to well‐balanced sensitivity and specificity using the Youden‐Index (Youden 1950). In a clinical context, it is also useful to maximize specificity in order to select patients who are very likely to develop a malignant infarction. For example, if a sNFL of > 643 pg/ml or sGFAP of > 108 ng/ml is detected and a malignant infarction is very likely, patients should preferably be treated in a neuro‐intensive care unit, transferred to a center with a neurosurgical department in a timely manner, or more frequent advanced bedside examinations such as pupillometry or transcranial ultrasound should be performed (Dowlati et al. 2021; Oliveira et al. 2022). Furthermore, if suitable (para)clinical findings are present, the decision to perform an early hemicraniectomy within 48 h or even ultra‐early hemicraniectomy within 24 h can be supported (Vahedi et al. 2007). It would also be conceivable to conduct a randomized controlled trial on ultra‐early hemicraniectomy in selected patients with large ischemic stroke and elevated biomarkers, which could further deepen the knowledge about the protective effect of hemicraniectomy in this setting. Similarly, it should be considered whether negative or neutral studies on other intensive care measures in the treatment of malignant infarction, such as mild therapeutic hypothermia or controlled normothermia, could only be effective in patients with elevated biomarker concentrations (Neugebauer et al. 2019; Greer et al. 2024). In contrast, if sNFL < 44 pg/ml or sGFAP < 2 ng/ml is detected, a more liberal diagnostic and therapeutic approach with preferred treatment on a stroke unit, extended CT monitoring intervals, accelerated weaning of analgesia or extubation strategies after thrombectomy or discontinuation of osmotic therapy can be considered to reduced potential harm of intensive care measures (Fandler‐Höfler et al. 2020; Moustafa et al. 2021).

The incorporation of sNFL and sGFAP measurement enhanced the capacity to predict malignant infarction for all combinations of two clinical or radiologically relevant parameters. This enhancement was observed in comparison to three of the four parameters of the well‐established malignant brain edema (MBE) score (Jo et al. 2017), which exhibits analogous AUC values compared to our prediction model. It is noteworthy that, even in comparison to the comprehensive model that incorporates all four significant predictors from multivariate analysis, the incorporation of a single serum biomarker enhanced the predictive value.

The existence of greater discrepancies in serum levels, increased AUC values, and enhanced prognostic model efficacy indicates that sGFAP possesses a superior prognostic value in comparison to sNFL. It is important to note that the blood draw was conducted 1–2 days after the AIS, with the simultaneous measurement of both blood‐based biomarkers. However, it should be noted that the release kinetics of both differ significantly. In particular, sGFAP shows a very early peak within 48 h after ischemic injury (Pujol‐Calderón et al. 2022; Ferrari et al. 2023), while sNFL increases after a prolonged period of time following the onset of symptoms and remains at a higher level for a longer time (Foerch et al. 2012; Wunderlich et al. 2006; Tiedt et al. 2018). The finding that two studies with smaller sample sizes were able to demonstrate a prognostic value of S100B (Foerch et al. 2004, Guimarães De Almeida Barros et al. 2023), another astrocytic protein, is also indicative of the consideration of a predominance of astroglial over neuronal markers in the prediction of malignant infarction. Nevertheless, neuron‐specific enolase, a reliable marker of neuronal damage and early stroke severity (Oh et al. 2003), was found to be a predictor of malignant infarction in large middle cerebral artery ischemic strokes (Zhang et al. 2018; Schaarschmidt et al. 1994). The established confounding of sNFL by renal function and age may provide a further possible rationale for the observed differences in the predictive values of the two serum biomarkers. However, the correlation for both parameters proved to be (very) weak and the multivariate analyses were adjusted for them.

Critically, it must be questioned whether the detection of elevated biomarkers in this study may not be specific enough to detect malignant infarct. Rather, it may be more generally a surrogate marker for predicting functional outcome, as has been shown in previous studies (Vollmuth et al. 2024, Barba et al. 2023). Another question relates to the current clinical consequence resulting from statistical significant benefit which can be achieved by measuring serum biomarkers. The combination of early signs of infarction with the severity of the clinical syndrome or the recanalization result after mechanical thrombectomy already allows a valid assessment of the further course of the disease. It is therefore important to note that although sNfL and sGFAP have independent predictive value for predicting malignant infarction, decisions about early interventions to treat malignant infarction should not be based solely on their measurement. Instead, they should be interpreted as a supplement to established clinical and imaging parameters.

Besides the study's prospective approach with quality‐assured data collection and the substantial size of the cohort with severe anterior circulation infarction, it is essential to acknowledge several limitations. Firstly, as previously mentioned, serum biomarker levels were measured only at a single point in time early after stroke. However, serial measurement to detect dynamic changes of serum biomarkers during the first days after a stroke could improve their predictive capacity. It appears that the increase between two standardized measurements, such as on admission and 24 h afterward, may be superior to a single determination (Correia et al. 2022). To account for individual release kinetics of different serum biomarkers, even further measurements, such as 48 h after the onset of symptoms, could be considered. On the other hand, in this study, blood sampling was performed a median of 36 h after the onset of stroke symptoms. This timeframe falls within the previously described optimal range for the determination of sGFAP peak levels (Pujol‐Calderón et al. 2022; Ferrari et al. 2023). In consideration of the release kinetics, it is important to acknowledge that the blood draw was conducted a median of four hours later in cases of malignant infarction. However, this finding does not account for the highly significant differences observed in biomarker measurements. Furthermore, multivariate analyses were adjusted for this parameter and showed no relevant impact on the results. Secondly, the primary focus of this study was clinical rather than advanced imaging focus, consequently, the assessment of malignant infarction was conducted through a combination of clinical and radiological endpoints (Huttner and Schwab 2009). Nonetheless, the resulting cohort appears to be highly representative, as previously mentioned. As an alternative, due to the crucial role of space‐occupying edema progression it would have been interesting to measure the edema volume as a paraclinical endpoint. In future studies, measurement of net water uptake (NWU) and its direct correlation with blood‐based biomarkers could strengthen our study results (Broocks et al. 2020). Additionally, further research should focus on the earlier collection of blood samples following an ischemic stroke, in addition to serial measurement (e.g., standardized sampling after 24 h).

## 5 Conclusion

The results of the present study demonstrate that sNfL and sGFAP are independent predictors of malignant infarction in severe anterior circulation ischemic stroke, although there is currently no clear additional clinical benefit compared to established forecast factors. The determination of these biomarkers appears, therefore, to be particularly useful in cases where imaging or clinical parameters do not provide clear results. Earlier and serial determination of biomarkers in a larger, multicenter follow‐up study would be necessary to improve prognostic significance and ensure the validation of cut‐off values. In addition, this would require the widespread introduction of a reliable point‐of‐care measurement, which is currently available for sGFAP but not for sNFL (Zylyftari et al. 2024; Oris et al. 2024).

## Author Contributions

**Dominik Lehrieder**: interpretation of results, statistical analysis, first draft of the manuscript. **Hermann Neugebauer**: conception and design of the study, study management and coordination, interpretation of results. **Alexander M Kollikowski**: collection of clinical, radiological and biochemical data, interpretation of results, revised the manuscript for intellectual content. **Patrick Oeckl**: biomarker analysis, interpretation of results, statistical analysis, revised the manuscript for intellectual content. **Felipe A Montellano**: interpretation of results, statistical analysis, revised the manuscript for intellectual content. **Lorenzo Barba**: biomarker analysis, interpretation of results, statistical analysis, revised the manuscript for intellectual content. **Peter U Heuschmann**: interpretation of results, statistical analysis, revised the manuscript for intellectual content. **Guido Stoll**: interpretation of results, revised the manuscript for intellectual content. **Markus Otto**: interpretation of results, revised the manuscript for intellectual content. **Mirko Pham**: interpretation of results, revised the manuscript for intellectual content. **Michael K Schuhmann**: interpretation of results, revised the manuscript for intellectual content. **Christoph Vollmuth**: conception and design of the study, study management and coordination, collection of clinical, radiological and biochemical data, interpretation of results, statistical analysis, first draft of the manuscript.

## Funding

CV, AMK and FAM were supported by the German Research Foundation (Deutsche Forschungsgemeinschaft, DFG), Project No. 413657723 (Clinician Scientist‐Programme UNION CVD), further CV was supported by the IZKF (interdisciplinary centre for clinical research), Z‐3BC/11. MKS was supported by the Hentschel Stiftung. The Wuerzburger Stroke Cohort was funded by the Deutsche Forschungsgemeinschaft (DFG, German Research Foundation)—Project No. 374031971 – TRR 240.

## Conflicts of Interest

PUH reports grants from German Ministry of Research and Education, German Research Foundation, European Union, Federal Joint Committee (G‐BA) within the Innovationfond, Charité–Universitätsmedizin Berlin, Berlin Chamber of Physicians, German Parkinson Society, University Hospital Würzburg, Robert Koch Institute, German Heart Foundation, University Göttingen (within FIND‐AF [A Prospective, Randomised, Controlled Study to Determine the Detection of Atrial Fibrillation by Prolonged and Enhanced Holter Monitoring as Compared to Usual Care in Stroke Patients] randomized, supported by an unrestricted research grant to the University Göttingen from Boehringer‐Ingelheim), University Hospital Heidelberg (within Registry of Acute Stroke Under Novel Oral Anticoagulants [RASUNOA]‐prime, supported by an unrestricted research grant to the University Hospital Heidelberg from Bayer, Bristol‐Myers Squibb, Boehringer‐Ingelheim, Daiichi Sankyo), grants from Charité–Universitätsmedizin Berlin (within MonDAFIS, supported by an unrestricted research grant to the Charité from Bayer), outside the submitted work. MO served as scientific advisor for Axon, Biogen, Roche and Fujirebio. All other authors have nothing to declare.

## Supporting information




**Supplementary file**: brb371333‐sup‐0001‐SuppMat.docx

## Data Availability

Anonymized data will be shared by request from a qualified investigator.
